# The Distinct Functions of Dopaminergic Receptors on Pain Modulation: A Narrative Review

**DOI:** 10.1155/2021/6682275

**Published:** 2021-02-22

**Authors:** Xia-qing Wang, Tahmineh Mokhtari, Yu-xuan Zeng, Lu-peng Yue, Li Hu

**Affiliations:** ^1^CAS Key Laboratory of Mental Health, Institute of Psychology, Beijing, China; ^2^Department of Psychology, University of Chinese Academy of Sciences, Beijing, China

## Abstract

Chronic pain is considered an economic burden on society as it often results in disability, job loss, and early retirement. Opioids are the most common analgesics prescribed for the management of moderate to severe pain. However, chronic exposure to these drugs can result in opioid tolerance and opioid-induced hyperalgesia. On pain modulation strategies, exploiting the multitarget drugs with the ability of the superadditive or synergistic interactions attracts more attention. In the present report, we have reviewed the analgesic effects of different dopamine receptors, particularly D1 and D2 receptors, in different regions of the central nervous system, including the spinal cord, striatum, nucleus accumbens (NAc), and periaqueductal gray (PAG). According to the evidence, these regions are not only involved in pain modulation but also express a high density of DA receptors. The findings can be categorized as follows: (1) D2-like receptors may exert a higher analgesic potency, but D1-like receptors act in different manners across several mechanisms in the mentioned regions; (2) in the spinal cord and striatum, antinociception of DA is mainly mediated by D2-like receptors, while in the NAc and PAG, both D1- and D2-like receptors are involved as analgesic targets; and (3) D2-like receptor agonists can act as adjuvants of *μ*-opioid receptor agonists to potentiate analgesic effects and provide a better approach to pain relief.

## 1. Introduction

Recently, the definition of pain has been revised by the International Association for the Study of Pain as “an unpleasant sensory and emotional experience associated with, or resembling that associated with, actual or potential tissue damage” [1, 2]. Vital to survival, pain functions as a protective alarm for an organism to identify and avoid possible life-threatening situations [3]. When pain lasts beyond normal tissue healing time (i.e., more than three months), it is known as chronic pain: a pathological condition with dramatic costs and suffering [4–6]. Pain is the integration of nociception with consciousness, feeling, and emotion and differs from nociception. It relies on the peripheral signaling pathway and involves several regions of the brain, including the thalamus, medial prefrontal cortex (mPFC), nucleus accumbens (NAc), periaqueductal gray (PAG), insula, somatosensory cortex, amygdala, and striatum [7–9].

The most widely prescribed analgesic drugs for pain relief, such as nonsteroidal anti-inflammatory drugs (NSAIDs), cyclooxygenase-2 (COX-2) inhibitors, and opioids, may have some adverse effects [10, 11]. For instance, a number of reports have proven that NSAIDs increase the risk of severe bleeding (especially gastrointestinal bleeding) and cardiovascular diseases (such as myocardial infarction and strokes), resulting in a higher rate of mortality [12, 13]. COX-2 selective inhibitors like celecoxib and rofecoxib show equivalent or superior analgesic and anti-inflammatory effects compared to conventional NSAIDs and carry a lower risk of gastrointestinal bleeding [14]. However, it has been demonstrated in animal models that inhibition of COX-2 activity might suppress bone healing [15, 16]. Although among analgesic agents, opioids are the most potent drugs for treating severe pain (e.g., cancer and perioperative pain), the efficiency of their long-term use for chronic pain is controversial [17, 18]. The critical problem is that long-term administration of opioids can cause addictive behaviors in patients (22.6%), further contributing to the opioid crisis [19, 20]. This evidence indicates that it is essential and urgent to investigate the analgesic efficacy of other known drugs or to develop new analgesic drugs with reduced side effects and abuse liability that target novel pathways.

A growing body of evidence from preclinical and clinical studies suggests that the dopamine (DA) system contributes to the pathology of the most chronic pain conditions [21, 22]. For instance, the studies with functional magnetic resonance imaging (fMRI) technique provided solid evidence suggesting that the mesolimbic dopamine circuit and pain modulation system are largely overlapped, including the ventral tegmental area (VTA), prefrontal cortex (PFC), amygdala, and nucleus accumbens (NAc) [23–25]. The functional connectivity between the aforementioned brain regions and their gray matter volumes was significantly different between chronic pain patients and healthy controls [26, 27]. And positron emission tomography (PET) used in the evaluation of patients with fibromyalgia syndrome demonstrated that synthesis and release of DA reduced in the presynaptic neurons [28]. Similar results were found in patients with back pain, indicating that altered brain DA function was associated with pain sensitivity and the affective state [29]. Besides this, several clinical studies have shown that the administration of levodopa, the precursor of DA, alleviates pain associated with Parkinson's disease in humans [30–32]. In animal models, injection of levodopa into the intrathecal (IT) space or some regions of the central nervous system (such as the NAc, dorsolateral striatum, and prefrontal cortex) had adequate analgesic effects [33, 34]. In general, these results indicate that the regulation of the dopaminergic system may be a novel strategy to manage pain effectively.

DA mainly acts on two subfamilies of DA receptors: D1-like (D1 and D5) and D2-like (D2, D3, and D4) receptors [35]. This classification is based on the biochemical and pharmacological properties of the receptors [36]. D1 subfamily receptors are coupled to G-protein alpha subunits (G_*α*s_ and G_*α*olf_), induce 3′,5′-cyclic adenosine monophosphate (cAMP) production, and activate protein kinase A (PKA) [37]. Members of the D2 subfamily are generally coupled to G_*α*i_ and G_*α*o_ subunits, inhibit the activity of adenylate cyclase, reduce the production of cAMP, and, subsequently, reduce PKA activity [38]. Although DA receptors are widely distributed in the brain, the densities of the receptor subtypes vary between different areas [39]. The D1 receptor is the most widespread protein in rat brains, whereas the D2 receptor is mainly distributed in specific regions such as the striatum, NAc, and some limbic regions [36, 40].

Previous reports have proven the role of the hypothalamic-spinal DAergic system on pain modulation, suggesting that the antinociceptive effects of DA are mediated by D2-like receptors, while the pronociceptive effects are mediated by D1-like receptors [41]. However, the available data fail to definitively elucidate the role of D1-like receptors on pain modulation and the effect of the central DAergic system on pain processing [41]. In the present review, we more comprehensively investigated the roles of DA receptor subtypes on pain modulation, especially D1 and D2 receptors in the central nervous system (CNS), to illustrate their potential analgesic features.

## 2. Spinal Cord

The spinal cord is the first relay station in the transmission of nociceptive information from the periphery to the brain. It has been proven that all types of DA receptors are present in the primary nociceptors and different laminars of the dorsal horns of the spinal cord and that D2 receptors are the most abundantly expressed [41, 42]. This indicates that DA functions in the modulation of pain signals by affecting presynaptic and postsynaptic neurons [41]. Numerous studies illuminated the contributions of D1- and D2-like receptors in the spinal cord during pain modulation. In cases of acute pain, intrathecal (IT) administration of DA or quinpirole (a D2/3 receptor agonist) was reported to increase the mechanical pain threshold, measured using a von Frey anesthesiometer. This analgesic effect was reversed by IT coadministration of D2, D3, and D4 antagonists [43, 44]. It was also demonstrated that IT administration of apomorphine or DA could enhance the thermal threshold, which was estimated by the tail-flick test [45–47] and the hot plate test [47]. These features were mimicked by administration of LY171555 (a D2-like receptor agonist), but not SKF-38393 (a D1/D5 receptor agonist), and inversed by D2 antagonists (e.g., *cis*-flupenthixol and sulpiride) [47]. For inflammatory pain induced by carrageenan, IT administration of LY171555, but not SKF-38393, rescued the thermal withdrawal latency (measured with Hargreaves apparatus) [48]. Similar findings were obtained from a neuropathic pain model of chronic constriction injury (CCI) of the sciatic nerve in which IT administration of levodopa decreased tactile and cold allodynia. This effect was blocked by sulpiride [34]. Furthermore, in a follow-up study, the same group found that lumbar IT injection of quinpirole produced short-term inhibition of responses to cold and tactile stimuli, which coincided with pain relief by levodopa administration in neuropathic pain [49].

Because DA can affect both exploratory and goal-oriented movements, the findings of these studies on the analgesic effects of DAergic drugs should be carefully interpreted [50]. These effects could potentially interfere with the results of pain-related behavioral tests, since the majority of measurements of the pain threshold utilize a withdrawal movement to a nociceptive stimulus [51]. For example, an effective analgesic dose of quinpirole administered systemically was proven to affect locomotion and exploratory activities of rats in an open-field maze [33]. While the intracisternal injection of quinpirole could successfully relieve formalin- and capsaicin-evoked trigeminal pain, a higher dose of quinpirole (20 nmol) had no effect on motor performance in the rotarod test (commonly used to evaluate motor coordination) [52]. Briefly, systematic administration of a high dosage of quinpirole may have affected the locomotor and exploratory activities of rats, while IT infusion of low doses had no significant effect on locomotion. Besides, the effects of D2 receptor agonists on locomotion might be strain-dependent, as shown in spontaneously hypertensive rat (SHR) and SLA16 isogenic (SHR.Lewis-*Anxrr16*; anxiety-related response #16) rat strains [53]. In summary, the dosage of drugs, route of administration, and strain of animals should be considered when investigating the possible analgesic effects of DAergic drugs.

*In vitro* studies have provided compelling evidence that DA is involved in pain signaling by modulation of intrinsic excitability and synaptic transmission of dorsal root ganglion neurons and spinal neurons [54]. For the first time, Tamae et al. used the whole-cell patch-clamp technique to record the excitability of spinal dorsal horn neurons and study the effects of DA receptor agonists. They found that bath application of DA hyperpolarized the membrane potential of substantia gelatinosa neurons and suppressed electrical stimulation action potential in the dorsal root. Quinpirole simulated a DA-induced outward current, which was not produced by SKF-38393 [43]. Moreover, activated D2 receptors in neurons of the superficial medullary dorsal horn inhibited the C-fiber-evoked action potentials of wide dynamic range neurons in the trigeminal spinal nucleus [52]. Consistent results were revealed in neuropathic pain, which reported that C-fiber-evoked action potentials in the spinal dorsal horn were dose-dependently ameliorated by spinal superfusion of quinpirole in both nerve-injured and sham-operated rats [55]. Collectively, D2 receptors may play an analgesic role in the spinal cord by reducing the amount of sensory inputs from nociceptors to the CNS (Table 1).

Interestingly, D1-like receptor agonists (SKF-83959 and SKF-81297) mimic the inhibitory effects of DA on slow ventral root potentials, which is attributed to responses evoked by C-fibers that reflect nociceptive transmission in the spinal cord. In addition, the inhibitory effects were attenuated by D1-like receptor antagonists (SCH-23390 and LE300) [56]. Taken together, there is widespread disagreement among *in vitro* studies about the role of D1-like receptors. However, *in vivo* investigations found that the D1 receptor had no significant analgesic effect in the spinal cord. These findings could be related to the lower affinity of DA for D1 receptors. Thus, only a few reports found that the activation of D1 receptors in the spinal cord could mimic the role of dopamine, while, in *in vivo* studies, not even a faint effect could be observed in behavioral testing.

## 3. Brain

The brain contains a high density of DA receptors in regions that functionally contribute to integrating the consciousness and emotion of pain, such as the ventral tegmental area (VTA), mPFC, NAc, PAG, and striatum [57, 58].

### 3.1. Ventral Tegmental Area (VTA)

The VTA, a part of the DAergic system, is composed of mesolimbic DA neurons that project to the NAc and mPFC [59, 60]. It is involved in various physiological functions such as pain processing and motivation [59]. For instance, the excitability of VTA DA neurons decreased significantly in CCI mice models. Optogenetic stimulation of VTA DA neurons produced analgesic effects [61]. In another study, voluntary wheel running was shown to produce hypoalgesia by reversing the inactivation of VTA DA neurons in a rat model with neuropathic pain [62]. Noxious footshocks are believed to inhibit the activities of DA neurons in the dorsal VTA, but physically excite the activities of DA neurons in the ventral VTA, suggesting that VTA DA neurons are heterogeneous in the processing of rewarding and aversive events [63]. Furthermore, a few reports have focused on the roles of different DA receptors in pain modulation. Pretreatment of VTA with both sulpiride [64] and SCH-23390 [65] could inhibit antinociception induced by intralateral hypothalamus (LH) microinjection of carbachol, obtained by the tail-flick test. The precise function of VTA DA neurons in the pain process is still unclear.

### 3.2. Nucleus Accumbens (NAc)

Functionally, NAc contributes to a wide range of reward-related behaviors [66]. As one of the two main projection target regions of VTA DAergic neurons (the other being the mPFC) [67], the NAc is a rostral telencephalic gray mass with a heterogeneous structure. Anatomically, it can be divided into the shell and core regions [68]. The shell is the outer region innervated by DAergic neurons and is closely related to the mesolimbic system. It has been identified as playing a profound role in motivation and pain modulation pathways [60, 69]. In contrast, the NAc core region, which connects to the caudate-putamen and striatum, is involved in goal-directed behaviors [70, 71]. Both D1 and D2 receptors were reported to be expressed in the whole NAc, while D3 receptors are selectively expressed in the shell region [69]. The past few decades have witnessed a drastic rise in the number of studies attempting to identify the analgesic effects of DA receptors in the NAc region. For example, stimulation of D2 receptors in the NAc was revealed to inhibit the persistent phase of formalin-induced nociception. The recommended dose of quinpirole did not produce overt behavioral changes, as shown in the rotarod treadmill test. However, the D1-selective agonist SKF-38393 had no significant effect on the nociceptive behavior induced by formalin [70]. Even so, a number of reports have demonstrated that blocking both D1- and D2-like receptors of the NAc attenuated analgesia induced by forced swim stress [72] and carbachol injection into the LH [73] in the formalin test, particularly in the late phase. And intra-accumbal administration of SCH-23390 and sulpiride dose-dependently prevented intra-VTA orexin-induced antinociception measured by the tail-flick test [74]. In addition, mRNA levels of D2 and D1 receptors both decreased in the NAc of animals with neuropathic injury, including spared nerve injury [75] and CCI [76].

Although the analgesic effects of D2 receptors in the NAc are more profound, blocking both D1- and D2-like DA receptors showed similar pharmacological effects [71, 72, 74]. The similarity of D1 and D2 receptors in the NAc may be attributed to their distinct DAergic innervations. The first relates to rewarding and pleasurable effects that act on primary D1 receptors of spiny neurons via direct pathways, and the second relates to aversive and negative effects that indirectly operate on spiny neurons, which is diminished by the activation of D2 receptors [77]. In other words, the activation of D1 receptors in the NAc probably weakens pain by enhancing the reward and pleasure effects, while D2 activation may reduce aversion to pain [78]. Compared to D1-like receptors in the NAc, D2-like receptors have a higher affinity to DA and are activated preferentially [79, 80]. However, further studies are needed to shed light on the potential mechanisms of D1- and D2-like receptors in pain modulation.

### 3.3. Prefrontal Cortex (PFC)

Studies over the past two decades have revealed the prominent role of the PFC in reward and pain modulation [81, 82]. As a region of the cerebral cortex located at the front of the frontal lobe, the PFC is a key structure contributing to critical brain functions such as memory, learning, attention, problem-solving, planning, and social behavior [83]. Recently, the important role of the DAergic signaling pathway from the VTA to the PFC on pain modulation has been proven [60, 84]. Anatomically, the PFC can be divided into the medial PFC (mPFC), dorsolateral PFC (dlPFC), ventrolateral PFC (vlPFC), orbitofrontal cortex, and caudal PFC [85]. Due to the potential pain modulating mechanisms of the DA system in the PFC, neuronal responses of the PFC were recorded using an extracellular recording unit in urethane-anesthetized rats. Applying a high-frequency stimulation (50 Hz, 250 *μ*A, 100 *μ*s square pluses, 30 s) to the VTA by a tungsten microelectrode (impedance 8-12 M*Ω*) suppressed nociceptive responses for an extended period in the rat PFC. Similarly, injection of a selective D2 receptor into the rat PFC produced long-lasting suppression of nociceptive responses, which was reversed by the blockade of D2 receptors. In contrast, the D1 antagonist/agonist was minimally effective in nociceptive responses [84].

The mPFC has a critical role in both reward and pain processing [86]. Projection of adrenergic neurons from VTA to mPFC (the DA inputs from VTA to mPFC) regulates the neural functions of mPFC (e.g., executive activities, excitability, and synaptic transmission) [87]. In chronic pain rodent models, the activity of neurons in the mPFC was reportedly reduced [88]. Optogenetic studies revealed that the activation of DA signaling from the VTA into the mPFC modulated hypersensitivity in mice with spared nerve injury neuropathic pain [89]. In summary, these studies suggest that the DA system in the mPFC may be a target for the relief of chronic pain.

DA modulation in the mPFC is also related to attention tasks for this region. Previous studies have proven the impairment of memory and attention by chronic pain [90]. In a rodent model, blocking the activity of D1 receptors in the mPFC impaired the attentional set-shifting task, whereas enhancing D1 receptor activity improved this performance [91, 92]. Based on these findings, clinical studies have shown that distraction tasks could decrease pain perception and relieve chronic back pain [93, 94]. Together, pain modulation of DA receptors in the mPFC may underlie distinct manners: D1 receptors tend to affect the cognitive aspect of pain (e.g., by regulating distraction), while D2 receptors may directly regulate the pain perception or nociceptive responses [83, 95]. Because of the complexity of the neuronal network, cellular receptor expression, DA concentration, and receptor affinity, it remains unclear as to how the DA system in the mPFC is involved in the pain modulation process.

### 3.4. Striatum

The dorsal striatum, which receives afferents from the sensorimotor cortex and DA-containing inputs from the pars compacta of the substantia nigra, has abundant D1 and D2 receptor expressions [96, 97]. Microinjection of a D1 antagonist (SCH-23390) or a D1 agonist (SKF-38393) into the dorsolateral striatum was reportedly not effective in formalin-induced nociception. Conversely, a D2 antagonist (eticlopride) could enhance the formalin-induced nociception, while a D2 agonist (quinpirole) exerted an opposite effect [98]. A similar result was found in neuropathic conditions in rats with unilateral ligation of the tibial and common peroneal nerves [99]. The antihypersensitive effect is induced by striatal D2 receptors and involved in inhibiting the impulse discharge of, presumably, pronociceptive neurons in the rostral ventromedial medulla, which is an important structure in descending pain modulation [100]. The antihypersensitive effect can be reversed by spinal administration of a D2 receptor antagonist [99]. Microinjection of quinpirole into the striatum inhibited the jaw opening reflex (JOR, an indicator of pain), evoked by tooth pulp stimulation, in a dose-dependent manner. This effect was blocked by haloperidol (a D2 receptor antagonist), whereas SKF-38393 left the JOR amplitude unaffected. Intrastriatal microinjection of quinpirole significantly reduced the responses of A*β*- and C-fiber afferents associated with inhibition of the JOR [101]. Thus, it is possible that striatal D2 receptors attenuate pain-related responses through final descending sensory pathways.

Notably, evidence from human studies also indicated that striatal D2 receptors are involved in the regulation of pain [102]. A previous PET study showed that healthy male volunteers with low D2 receptor availability in the right putamen exhibited a high cold-pain threshold, while a low heat-pain modulatory capacity was associated with low D2 receptor availability in the left putamen [103]. Similarly, D2 receptor availability in the left putamen of patients with chronic orofacial pain increased [104]. Such results concur with experimental animal studies. More specifically, a high level of synaptic DA can lead to low availability of D2 receptors, a high cold-pain threshold, and a low response capacity to conditioning stimulation [103]. In other words, the high availability of D2/D3 receptors predicts a low synaptic DA concentration and a high capacity of recruiting pain inhibitory circuitry [96]. In addition, previous reports also demonstrated that striatal D2 receptors may influence pain-related responses not only through descending modulation of sensory pathways but also through supraspinal action on nonsensory factors [96, 97, 102]. For instance, the responses of the subject's attitude toward pain were negatively correlated with baseline striatal D2/D3 receptor availability [105]. Thus, subjects with high availability of striatal D2/D3 receptors, probably indicating low extracellular endogenous DA levels, rate the same stimulus as more painful than subjects with low striatal D2/D3 receptor availability [106].

### 3.5. Periaqueductal Gray (PAG)

The midbrain PAG, a region full of opioid receptors, plays a significant role in the modulation of nociception. A subpopulation of DAergic neurons (A10-dc) known to be involved in the modulation of endogenous pain exists in the ventrolateral PAG (vlPAG), which projects locally within the PAG or to forebrain regions [107, 108]. Electrical stimulation of the PAG led to pain relief in animal models [109, 110]. Also, impairment of dopaminergic neurons of the PAG reduced antinociception induced by opioids [111]. In another study, enhanced levels of tyrosine hydroxylase following induction of CCI resulted in an enhanced level of DA in the PAG [112].

Microinjection of (–) apomorphine (DA agonist) into the ventral PAG (vPAG) was shown to increase the latency to lick the hind paw in the hot plate test in a dose-dependent manner. This effect was blocked by pretreatment with eticlopride (D2 antagonist), but not SCH-23390 (D1 antagonist). Apomorphine infusion into the outside of the vPAG had no marked analgesic effect [113]. In contrast, the findings of another study showed that administration of D1 receptor antagonist SCH-23390 in PAG dose-dependently attenuated analgesia induced by opiates (e.g., heroin and morphine). The effect was observed by the behaviors integrated supraspinal response (examined by hot plate test), but not the simple spinal reflex (examined by the tail-immersion test) [111]. Additionally, several studies have found that injection of both D2-like and D1-like receptor antagonists (raclopride and SCH-23390, respectively) into the PAG reduced antinociception induced by the activation of *μ*-opioid receptors [114]. Selective activation of D2-like receptors within the PAG significantly reduced allodynia, which was also blocked by GABAA receptor agonist (muscimol), opioid receptor antagonist (naloxone), and D2-like receptor antagonist (raclopride). Although the analgesic effect induced by activating D1-like receptors in the PAG was tiny and transient, it enhanced the antinociceptive effects of D2-like receptors [114]. Notably, treatments with all drugs had no significant influence on the locomotion of rats observed in open-field tests. Overall, these findings indicate that the activation of D2 receptors may induce the antinociceptive effects directly, while the activation of D1 receptors may participate synergically in the opioid-induced and D2-induced antinociception [111, 114].

In short, D2-like receptors may exert a higher analgesic potency, but D1-like receptors act in different manners with distinct mechanisms in the mentioned regions (Table 2). However, more studies should be designed to thoroughly investigate the role of DA receptors in antinociception and the underlying mechanisms.

## 4. Discussion and Conclusions

Complaints about acute and chronic pain reflect that pain management is poorly served by existing treatments. Developing novel analgesics with superior efficacy, diminished adverse effects, and lower abuse liability is urgent. Previous studies showed that the DAergic system plays a critical role in pain modulation [21, 115], suggesting that targeting DA receptors may be a novel treatment strategy for chronic pain. Therefore, we reviewed the role of DA receptor subtypes in the pain processes, particularly D1 and D2 receptors, throughout the central nervous system.

The evidence from studies on the spinal cord and brain cortex consistently indicated that more potent analgesic effects are related to D2 receptors, while the role of D1 receptors on pain modulation varies between different parts of the nervous system. Firstly, D1 receptors are mainly located postsynaptically, while D2 receptors are in both post- and presynaptic regions. Presynaptic DA receptors are characterized by higher sensitivity (5- to 100-fold) than those located in postsynaptic parts [116]. Therefore, compared to the D1 receptors, D2 receptors have a higher affinity for DA and require lower concentrations of DA to be activated. Secondly, D2 receptor signaling has an inhibitory effect, and the activation of D2 receptors decreases a neuron's firing rate, resulting in the network requiring more stimuli to initiate DA transmission [21]. For instance, in the spinal cord, D2 receptors play an analgesic role by reducing the intensity of the sensory input, like depressed C-fiber-evoked field potentials [55]. However, D1 receptors mediate neuronal excitation, which relies on the complex neuronal network between regions and a higher DA level.

In addition to the more potent analgesic efficacy of D2 receptors, D1 family receptors may play a more important role than D2 family receptors in mediating the facilitation of abuse-related intracranial self-stimulation (ICSS; one experimental procedure evaluated abuse-related effects of drugs) [117]. High-efficacy D1 agonists SKF-82958 and A77636 produced facilitation of abuse-related ICSS depending on dosage and time. Lower efficacy D1 ligands and all D2/3 ligands failed to facilitate ICSS at any dose or pretreatment time. Besides, D2 receptor agonists are long prescribed and well tolerated, which are excellent features of clinical medicine [55].

Notably, although mounting evidence has revealed the antinociceptive effects of D2-like receptors, their analgesic efficacy is not as high as other drugs [55, 115]. Fortunately, a few studies have suggested that D2 receptors establish complex interactions with opioid receptors and could potentiate the analgesic effects of *μ*-opioid receptor agonists. For instance, coadministration of 1 *μ*mol/L through spinal infusion was insufficient to alter potentials evoked by electrical C-fiber stimulation but could dramatically enhance the potential inhibition effects of DAMGO ([D-Ala^2^,N-MePhe^4^,Gly-ol]-enkephalin; a *μ*-opioid receptor agonist) and reduced its half-maximal inhibitory concentration (IC50) by 2-fold in a rat model of peripheral nerve injury [55]. Furthermore, the IT administration of quinpirole in lower doses remarkably augmented the antinociception effects of DAMGO (8-fold) in both inflammatory and neuropathic models of pain, assessed by mechanonociception and thermonociception behavioral tests [118]. Systemic administration of *R*-VK4-40, a highly selective D3 receptor antagonist, produced mild antinociceptive effects without altering locomotion, as observed in the open-field test or rotarod locomotor performance. In rats, this substance diminished the rewarding potency of oxycodone (the most commonly abused prescription opioid) and mitigated its tolerance and dependence without compromising its analgesic effects [119]. Thus, targeting the D2-like receptors may have the potential to enhance the analgesic property and alleviate the adverse effects of opioids.

Moreover, as previous reviews focused more on the role of the two subfamilies of DA receptors in the spinal cord and hypothalamus, the present review provides a more comprehensive summary on this topic by including the spinal cord and different brain regions. Indeed, some other regions (e.g., the hippocampus and amygdala), which are also involved in pain modulation, were excluded from the review due to the shortage of relevant studies, making it difficult to deduce a reliable conclusion. Moreover, even though we summarized the mutual promotion between the D2 receptor agonists and the opioids, whether chronic exposure to coadministration of D2-like receptor agonists and opioids could decrease the side effects of long-term opioid use remains unknown.

In summary, due to the vital role of the dopaminergic system on pain modulation, exploiting the multimodal analgesic regimens that target different DA receptors attracts more attention. Thus, we reviewed relevant studies to clarify the potential analgesic features of the DA receptor subtypes, especially D1 and D2 receptors, and summarized as follows: (1) D1 receptors act in different manners with distinct mechanisms in several regions, including the spinal cord, striatum, nucleus accumbens (NAc), and periaqueductal gray (PAG); (2) compared with D1 receptors, D2 receptors may exert a higher analgesic potency. Considering the superadditive or synergistic interactions between the D2 receptors and the opioid receptors, the agonist of D2 receptor may work as an adjuvant to potentiate the analgesic effect and reduce the side effects of opioids [55, 118].

## Figures and Tables

**Table 1:** The role of DA receptors on pain modulation in the spinal cord.

Authors	Drugs	Models	Measurements	Main results
Almanza et al. [44]	Quinpirole (D2/3 agonist)	Acute pain	Von Frey Hargreaves apparatus	(i) The activation of dopamine D2 receptors increased mechanical threshold
Barasi and Duggal [45] Jensen and Smith [46] Liu et al. [47]	LY171555 (D2 agonist) SKF38393 (D1/D5 agonist)	Acute pain	Tail-flick test Hot plate test	(i) The D2 agonist mimicked the analgesic effect of DA, but the D1 agonist did not
Gao et al. [48]	LY171555 SKF38393	Inflammatory pain induced by carrageenan	Hargreaves apparatus	(i) The D2 agonist rescued the thermal withdrawal latency, but the D1 agonist did not
Cobacho et al. [49]	Levodopa Sulpiride (D2 antagonist)	Neuropathic pain induced by chronic constriction injury	Tactile and cold allodynia test	(i) Levodopa decreased the tactile and cold allodynia, which was blocked by the D2 antagonist
Tamae et al. [43]	Quinpirole SKF 38393	Acute pain	Von Frey filament whole-cell patch-clamp technique	(i) The D2 agonist simulated the analgesic effect of DA at both behavioral and electrophysiological levels, but the D1 agonist did not
Lapirot et al. [52]	Quinpirole Sulpiride	Acute pain	Unitary extracellular recordings Facial capsaicin and formalin test	(i) The activation of D2 receptors inhibited both formalin- and capsaicin-evoked pain behaviors and the C-fiber-evoked action potential firing

**Table 2:** The role of DA receptors on pain modulation in the brain.

Authors	Brain regions	Models	Drugs	Measurements	Main results
Moradi et al. [64]	VTA	Acute pain	D1 antagonist SCH23390 D2 antagonist sulpiride	Tail-flick test	(i) Blockage of the D1 and D2 receptors had a similar effect, as inhibiting the antinociception induced by carbachol
Taylor et al. [70] Siahposht-Khachaki et al. [73] Yazdi-Ravandi et al. [74] Martikainen et al. [29]	NAc	Acute pain and neuropathic injury (SNI and CCI)	D1 antagonist SCH23390 D1 agonist SKF38393 D2 antagonist sulpiride D2 agonist quinpirole	Formalin test Tail-flick test	(i) Neuropathic injury decreased the mRNA levels of both D2 and D1 receptors (ii) The blockade of D1- and D2-like receptors showed the similar pharmacological effects (iii) The activation of the D2 receptors had more profound analgesic effects
Sogabe et al. [84] Granon et al. [92] Fletcher et al. [91]	PFC	Acute pain In vitro	The same as above	Electrophysiological recording Attentional set-shifting task	(i) The D1 receptors tend to affect the cognitive aspect of pain, like via distraction (ii) The D2 receptors may directly regulate the pain perception or nociceptive responses (iii) The activation of the D2 receptors had more profound analgesic effects
Magnusson and Fisher [98] Ansah et al. [99] Saunier-Rebori and Pazo [101]	Striatum	Inflammatory pain	D1 antagonist SCH23390 D1 agonist SKF38393 D2 antagonist eticlopride D2 agonist quinpirole	Formalin test Jaw opening reflex	(i) The activation of the D2 receptors had antihypersensitive effect, but not D1 receptors
Hagelberg et al. [103, 104]	Striatum	Clinical chronic orofacial pain		PET	(i) Healthy male volunteers with a low D2 receptor, while D2 receptor availability of patients increased, which predicted a low synaptic DA concentration and a high capacity of recruiting pain inhibitory circuitry
Ben-Haim et al. [109] Li et al. [107] Tobaldini et al. [114]	PAG	Acute pain	DA agonist apomorphine D2 antagonist eticlopride D1 antagonist SCH-23390 Opiate heroin and morphine	Hot plate test Tail-flick test Mechanical paw-withdrawal test	(i) The analgesic effect of apomorphine was blocked by D2 antagonist, but not by D1 antagonist (ii) Blockage of the D1 and D2 receptors had a similar effect, as reducing the antinociception induced by the opioids (iii) The activation of D1 receptors could enhance the opioid- and D2-induced antinociception

## Data Availability

All the researches involved in the present review are available in standard databases such as Web of Science, PubMed, and MEDLINE.
